# Role of the Mitochondrial E3 Ubiquitin Ligases as Possible Therapeutic Targets in Cancer Therapy

**DOI:** 10.3390/ijms242417176

**Published:** 2023-12-06

**Authors:** Jacopo Di Gregorio, Martina Appignani, Vincenzo Flati

**Affiliations:** Department of Biotechnological and Applied Clinical Sciences, University of L’Aquila, 67100 L’Aquila, Italy; jacopodigregorio@gmail.com (J.D.G.); martina.appignani@student.univaq.it (M.A.)

**Keywords:** ubiquitin ligase, cancer, mitochondria

## Abstract

Ubiquitination is a post-translational modification that targets specific proteins on their lysine residues. Depending on the type of ubiquitination, this modification ultimately regulates the stability or degradation of the targeted proteins. Ubiquitination is mediated by three different classes of enzymes: the E1 ubiquitin-activating enzymes, the E2 ubiquitin-conjugating enzymes and, most importantly, the E3 ubiquitin ligases. E3 ligases are responsible for the final step of the ubiquitin cascade, interacting directly with the target proteins. E3 ligases can also be involved in DNA repair, cell cycle regulation and response to stress; alteration in their levels can be involved in oncogenic transformation and cancer progression. Of all the six hundred E3 ligases of the human genome, only three of them are specific to the mitochondrion: MARCH5, RNF185 and MUL1. Their alterations (that reflect on the alteration of the mitochondria functions) can be related to cancer progression, as underlined by the increasing research performed in recent years on these three mitochondrial enzymes. This review will focus on the function and mechanisms of the mitochondrial E3 ubiquitin ligases, as well as their important targets, in cancer development and progression, also highlighting their potential use for cancer therapy.

## 1. Introduction

Among the post-translational modifications, ubiquitination is one of the most extensively studied. Ubiquitination consists of the attachment of ubiquitin, a small protein of 76 amino acids in length, on specific lysine (K) residues of the targeted protein [1]. The attached ubiquitin can undergo further ubiquitination, and depending on the specific lysine residue on the ubiquitin sequence (K6, K11, K27, K29, K33, K48 and K63), different poly-ubiquitin chains can be formed and are named after the specific lysine residue [2].

The different ubiquitination types lead to different cell fates: among the most important, K48 ubiquitination brings the tagged protein to the proteasome for degradation, K63 ubiquitination is related to various cellular processes such as endocytic trafficking, inflammation and DNA repair, K11 is implicated in mitotic regulation and endoplasmic-reticulum-associated degradation (ERAD), K6 is involved in DNA repairs and DNA modifications such as methylation, and K27 is involved in mitochondrial DNA repair [3].

The process of ubiquitination is a multi-reaction cascade and requires three different classes of enzymes, each one catalyzing a different step of the reaction. The enzymes are classified into E1 (ubiquitin-activating enzyme), E2 (ubiquitin-conjugating enzymes) and E3 (ubiquitin ligases). The E1 activating enzyme activates ubiquitin at its C-terminus in an ATP-dependent manner: this enables ubiquitin to be transferred by the E2 conjugating enzymes to the E3 ligases that are capable of binding both the E2 and the specific substrate [4,5].

The third step, the most important of the cascade, is called “ligation” and is mediated by an E3 ubiquitin ligase that transfers ubiquitin from the E2 to the specific substrate. E3 ligases mediate the specificity of the ubiquitination reaction by recognizing specific structures and motifs on the targeted protein. Outside of the catalytic core, the rest of the structure of the E3 ligases is responsible for recognizing the specific substrate. Paired with the specific localization of those enzymes, which allows ubiquitination of the co-localized substrates, this creates a vast network of post-translational regulation, resulting in protein quality control, degradation of overproduced proteins and their activation and localization [5].

To allow ubiquitination and control of so many different substrates, in the human genome, there are more than a thousand different E3 enzymes, divided into four classes based on the structure of their catalytic domain: HECT (homologous to the E6AP carboxyl terminus), RING (Really Interesting New Gene), U-Box and RBR (RING-IBR-RING, a hybrid between HECT and RING). The four classes of E3 enzymes are responsible for different cellular functions [5].

Each E3 ligase can recognize and interact with several substrates. Depending on their substrates, as well as the different lysine residues that are ubiquitinated, E3 ligases can then be involved in a plethora of cellular and physiological processes. Moreover, alteration in the function of the E3 ligases can lead to several pathological scenarios [6]. In fact, the loss of activity of an E3 ligase would result in the accumulation of its targets, which may lead to pathology. On the other hand, excessive activity of one or more E3 ligases would mean complete degradation of their targets and ultimately their loss of function [6].

Alterations and mutations of a wide number of E3 ligases have been linked to different neurological diseases, such as encephalopathy, ataxia or Parkinson’s disease [7,8].

Furthermore, dysregulation of E3 ligases, both in the form of hyperactivation and downregulation, has been associated with cancer progression, as well as with evasion from cell death, and metastasis [9].

Another common trait in many tumors is the alteration of mitochondria: this leads not only to metabolic changes in the neoplastic cells but also to an alteration in the production of reactive oxygen species, as well as in their signaling, increased proliferation and changes in the interactions with the tumor microenvironment [10].

When merging these two concepts, it becomes clear that understanding the alterations in the mitochondrial E3 ubiquitin ligases may be relevant for finding new strategies for cancer therapy. There are only three E3 ligases specifically localized in the mitochondria: MARCH5, RNF185 and MUL1, also called collectively “mito E3 ligases”. These enzymes are all RING finger-type ligases and are capable of ubiquitinating also cytosolic targets.

Despite their basic functions being canonically related to cellular homeostasis, the mito E3s are found dysregulated in several cancers. This review will highlight the different roles of MARCH5, RNF185 and MUL1 in cancer, focusing on their functions as potential oncogenes or tumor suppressors and defining a possible use for therapy. Despite other reviews on E3 ligases present in the literature, to our knowledge, this is the first one to specifically focus on the only three localized in the mitochondria. Although this excludes other E3 ligases that may represent important targets for cancer therapy [7], resulting in a potential limitation for this review, we believe that focusing on the mito E3 ligases would be useful to pinpoint the connections between mitochondrial alterations (such as increased mitophagy, fission and fusion, ROS and metabolite production) in cancer and derangements of the mito E3 ligases activity (inevitably affected by mitochondrial alterations). Understanding the oncogenic or tumor suppressor activity of the mito E3s could be an additional tool in order to expand the knowledge about the role of mitochondria in cancer.

## 2. MARCH5

The Membrane-Associated RING-CH Protein V protein (MARCH5) is located in the outer mitochondrial membrane (OMM). Also known as MITOL and RNF153, it was first identified in 2006 and associated with mitochondrial dynamics: in fact, MARCH5 targets for K-48 ubiquitination the mitochondrial proteins Mitofusin 2 (MFN2) and Dynamin Related Protein 1 (DRP1), thus contributing to the regulation of mitochondrial fission and fusion [11]. Other well-established targets of MARCH5 include the DRP1 receptor MiD49 [11], the mitochondrial quality control protein FUN14-Domain-Containing Protein 1 (FUNDC1) [12], the retinoic acid-inducible gene-I (RIG-I) receptor [13], the ER-associated endoribonuclease IRE1α [14] and the mitochondrial antiviral signaling (MAVS) protein [15]. MARCH5 has thus been canonically involved in mitochondrial protein quality control, mitophagy, ER stress and unfolded protein response (UPR), innate immune response and prevention of cell senescence. Via ubiquitination of MFN2, MARCH5 mediates the physical interaction between mitochondria and the ER, allowing the exchange of lipids and calcium ions at the membrane contact site. Depletion of MARCH5 has been related to the disruption of the membrane contact site and the aggravation of derangements caused by progressive diseases [16]. Due to its role in mitochondrial quality control, MARCH5 has been associated with neurodegenerative diseases, where damaged proteins may translocate to the mitochondria. Consequently, this connects MARCH5 also to senescence and aging. This suggests the use of MARCH5 activators, such as berberine, as anti-aging drugs [17].

MARCH5 is regulated by auto-ubiquitination [16], targeting itself for proteasomal degradation to maintain correct mitochondrial homeostasis. In addition, it can be negatively regulated by the mir-30a microRNA [17].

In the context of cancer, MARCH5 tends to act as an oncogene, with different mechanisms that depend on the cellular context and are characterized by the specific targets of the ligase.

In fact, MARCH5 can be found upregulated in breast cancer (BC): high levels of the ligase have been found in several BC cell lines, as well as in cultures derived from BC patients [18], mainly due to a downregulation of mir-30a. MARCH5 expression in the context of BC is related to poor prognosis [19]. Increased MARCH5 expression results in lower levels of its targets such as DRP1 and MFN2 and altered autophagy, ultimately leading to dysfunctional mitochondria, increased reactive oxygen species (ROS) production and increased aggressiveness and metastatic potential. This was proven by knocking down MARCH5 in a model of BC cells that led to reduced cell growth and metastatic ability. This was also shown in vivo in an MDB-MB xenograft on MARCH5 KO mice where it caused slower tumor growth and a smaller number of metastases [19].

In addition, inhibition of MARCH5 in BC results in increased sensitivity toward apoptosis [18,19]. These reports establish the oncogenic potential of MARCH5 in BC and suggest an inhibition strategy by targeting the ligase for therapeutic purposes.

Similar mechanisms are found in ovarian carcinoma (OC): studies of tissue samples of ovarian cancer highlighted higher levels of MARCH5 when compared to healthy controls. Also in this case, the silencing of MARCH5 resulted in reduced growth and invasion. These observations were confirmed also in a nude mouse model. In fact, the role of MARCH5 in OC progression and invasion involves an induction of the autophagic process mediated by Transforming Growth Factor Beta 1 (TGFβ1), which is directly enhanced by MARCH5 overexpression. This upregulates, in a TGFβ1-dependent manner, the levels of the protein LC3B, a marker of autophagy activation. Together with a direct, MARCH5-dependent, induction of the autophagy-related protein ATG5, the autophagic process is increased, thus leading to cancer progression. Furthermore, when MARCH5 is silenced in this cellular context, autophagy is inhibited, and cancer progression is slowed. The mechanism seems to be dependent on MARCH5 RNA, which increases the mRNA of ATG5 and SMAD2. Moreover, the canonical TGFβ/SMAD2 signaling pathway has an inhibitory effect on mir-30a, thus creating a positive feedback loop for MARCH5 activation in OC [20].

Furthermore, the knock-down of MARCH5 in colorectal carcinoma and melanoma mouse models has been associated with reduced tumor growth and increased anti-tumor immunity. Indeed, MARCH5 is transcriptionally induced by the immune checkpoint PD-1, ubiquitinating and degrading the Common Cytokine Receptor γ chain and leading to cancer growth. Moreover, inhibition of MARCH5 synergizes with PD-1 blockade in inhibiting tumor growth in colorectal cancer and melanoma [21].

The oncogenic role of MARCH5 in melanoma is exerted also with a different mechanism that involves the endoplasmic reticulum (ER) stress resistance. In the context of melanoma, this is relevant because the activation of ER stress may represent a valid therapeutic target for melanoma treatment [22,23,24]. However, mitochondria can reduce the apoptosis induced by ER stress. ER stress resistance can be induced by dysregulating mitophagy, inducing mitochondrial fission and inhibiting the expression of MFN2, thus making melanoma cells insensitive to this kind of therapy [25].

As seen in an in vitro model, melanoma cells degrade MFN2 to achieve ER stress resistance: the mechanism for this degradation relies solely on MARCH5, as only the silencing of this ligase was able to restore the protein levels of MFN2 in melanoma. In this cellular context, MARCH5 is hyperactivated by the transcription factor X-box binding protein 1 (XBP1) and degrades MFN2. This molecular axis between XBP1, MARCH5 and MFN2 could be targeted to use ER stress to reduce cancer progression in melanoma [25].

In this regard, these studies are consistent with the ongoing research on MFN2 where it is identified as a tumor suppressor gene. As seen in hepatocellular carcinoma (HCC), MFN2 mediates p53-induced cell death [26], apoptosis induction via Bax co-localization [27] and increased Ca++ intake from the ER to the mitochondria, leading to cell cycle arrest [28]. In breast and lung cancer, MFN2 inhibits cell survival via Akt and Erk downregulation [28,29]. Depletion of MARCH5 in those cancers could help raise the levels of MFN2 and strengthen its effect. Moreover, monitoring MARCH5 levels in the same cancer could be a prognostic indicator because an increase in the levels of MARCH5 would negatively reflect on the protein levels of MFN2, and vice versa.

The potential oncogenic role for MARCH5, which results in the regulation of its canonical targets, has been investigated in several other cancer cell lines (prostate cancer, lung carcinoma and osteosarcoma). Deletion of MARCH5 has been in fact associated with increased sensitivity to BH3 mimetics-induced cell death, with an increase in MCL1 levels and activation of NOXA. [30]. Further studies on this mechanism have shown that MARCH5, other than ubiquitinating MCL-1 for proteasomal degradation, directly controls the turnover of the MCL1/NOXA complexes, as both proteins increase in the absence of MARCH5. In addition, the depletion of MARCH5 increases the mitotic cell death induced by antimitotic drugs [31].

This regulation of MCL1 by MARCH5 could be exploited in other cancer scenarios, where MARCH5 has not yet been investigated and where the ligase may have a tumor suppressor role, despite opposite results in other cancers [32]. MCL1 overexpression can indeed lead to an unbalance between pro-apoptotic and anti-apototic factors. This is the case of multiple myeloma or leukemia models, where MCL1 is increased and thus often targeted for therapeutic purposes [33,34]. In these cancers, its overexpression is a poor prognostic marker. A similar role for MCL1 has been found in lung cancer, where MCL1 also activates the Akt signaling [35,36], as well as in colorectal cancer, where MCL1 has been associated with metastasis [37].

**Figure 1:**
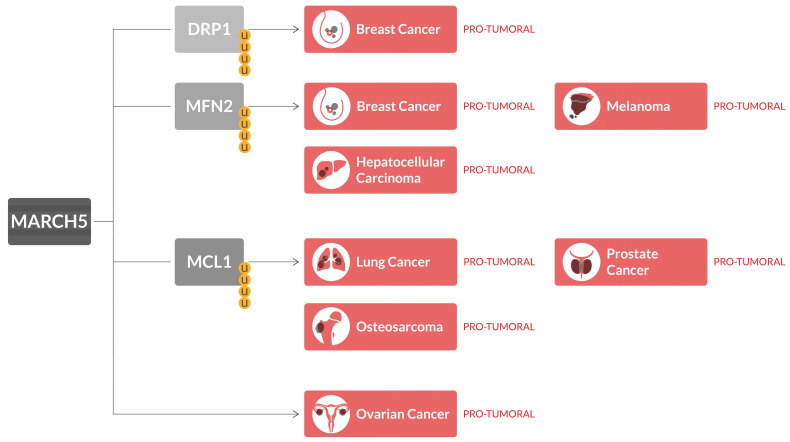
MARCH5 in cancer. Overexpression of MARCH5 is related to cancer progression, as ubiquitination (shown as yellow U in the figure) and degradation of its targets DRP1 and MFN2 are related to breast cancer, melanoma and hepatocellular carcinoma growth and development, while ubiquitination of MCL1 is associated with lung cancer, prostate cancer and osteosarcoma. Moreover, MARCH5 is upregulated in ovarian cancer.

In those types of cancer, MARCH5 may act as a tumor suppressor by inhibiting MCL1 overexpression, and a therapeutic strategy involving the use of BH3 mimetics could be paired with MARCH5 induction, in order to achieve cancer regression (Figure 1).

## 3. RNF185

RING Finger Protein 185 (RNF185) is located in the outer mitochondrial membrane, with its RING domain facing the cytosol. It was first identified in 2013, and it was first associated with ERAD, the process by which the unfolded proteins that accumulate in the ER are transported to the cytosol for degradation by the proteasome [38]. Directly or indirectly, RNF185 regulates several cell processes such as autophagy, immune response and endoplasmic reticulum protein degradation [38,39,40]. RNF185 regulates autophagy by stimulating LC3II accumulation and autophagosome formation in human cells. More specifically, RNF185 regulates selective mitochondrial autophagy, thus modulating mitochondrial homeostasis [40].

RNF185 regulates innate immune response by binding and regulating, through ubiquitination, the cyclic GMP-AMP synthase (cGAS) which is involved in the activation of the stimulator of interferon genes (STING) and innate immune response [39]. RNF185 deregulation is involved in several diseases. As shown by the reviewed studies below, RNF185 has been shown to act as a tumor suppressor, and thus, its lack of function is reflected in the development of cancer.

RNF185 is downregulated by the long non-coding RNA (lncRNA) RNF185-AS1. This has been found significantly upregulated in the hepatocellular carcinoma (HCC) serum of HCC patients, and high levels of RNF185-AS1 were found in HCC cells. It promotes HCC cell proliferation, epithelial–mesenchymal transition (EMT), migration and invasion while RNF185-AS1 knock-down inhibits the same processes. The invasive phenotype has been found to be mediated by the mR-221-5p/integrin β5 axis, and thus, its targeting may hold the promise for a possible therapeutic strategy in HCC [41,42,43].

RNF185-AS1 has also been found highly overexpressed in papillary thyroid carcinoma (PTC). This high expression has been found associated with larger tumor size, lymph node metastasis and advanced cancer stage in PTC patients. The mechanism of action is mediated by the downstream miR-429/lipoprotein-receptor-related protein (LRP4) axis. RNF185-AS1 silencing has been found capable of impeding the proliferation, migration and invasion of the cancer cells in vitro and to reduce tumorigenesis in vivo. Thus, the RNF185-AS1/miR-429/LRP4 axis may represent a potential target for the development of therapeutic strategies in PTC [44].

Downregulation of RNF185 expression has been found to correlate with prostate cancer progression and metastasis, and when RNF185 is reduced, the wound healing and cellular movement pathways were the most significant that were found upregulated. The deregulations were implicated with EMT and the acquisition of the migration phenotype. Collagen type III alpha 1 chain (COL3A1) has been identified as the main mediator of RNF185’s ability to promote the migration phenotype in prostate cancer cells as COL3A1 inhibition was capable of attenuating the migration and metastasis of the cancer cells [45]. Thus, the replenishment of RNF185 or the targeting of COL3A1 may be considered as possible strategies for prostate cancer therapy.

COL3A1 overexpression has also been found to be associated with poor prognosis and chemotherapy resistance in lung cancer [46], and it has been reported to mediate drug resistance in osteosarcoma [46]. On this basis, it can be hypothesized that RNF185, in a COL3A1-dependent manner, may act as a tumor suppressor in a broad spectrum of cancers; consequently, it may be targeted for the development of therapeutic strategies for different tumors.

Additionally, as mentioned earlier, RNF185 has been found to be among several E3 ubiquitin ligases that are significantly upregulated by endoplasmic reticulum (ER) stress and are involved with ERAD [47]. RNF185 is also part of an ER membrane complex together with the ubiquitin-like domain containing proteins TMUB1/2 and TMEM259/Membralin and exerts the quality control functions of ER membrane proteins and mediates their degradation [48]. Among them, there is Lanosterol 14-alpha demethylase (cytochrome P450(51) or CYP51A1TM. This is an enzyme that catalyzes one of the key steps in cholesterol biosynthesis [49]. Cholesterol modulates pathways involved in neoplastic transformation and tumor progression. Thus, CYP51A1TM could be targeted in order to interfere with cholesterol metabolism in cancers. In fact, CYP51A1 inhibition leads to the induction of apoptosis in cancer cells [50]. A further possible strategy to inhibit CYP51A1TM indirectly could be represented by the modulation of RNF185 activity, such as via gene therapy, for those cancers where it is underexpressed (i.e., HCC, PTC, prostate carcinoma).

Cystic fibrosis transmembrane conductance regulator (CFTR) is responsible for cystic fibrosis (CF) when it is mutated. Cystic fibrosis is a progressive, genetic disease that affects the lungs and other organs [51,52,53]. CFTR is a target of RNF185, in synergy with RNF5 (RNF185 is an RNF5 homolog), for co-translational degradation. In fact, RNF185 controls the stability of CFTR and the CFTRΔF508 mutant [54]. Thus, RNF185 and RNF5 are possible therapeutic targets for cystic fibrosis. Nevertheless, CF is a disease that affects other tissues, such as the gastrointestinal (GI) tract, other than the lungs. In fact, the CFTR loss of function, following its downregulation or mutation, has been associated with carcinogenesis of the GI tract and, in particular, with colorectal cancer (CRC), which is increased by 6-fold [53,54]. Thus, RNF185 could play an oncogenic role in gastric cancer (GC). This is a common malignant cancer and, when in an advanced state, is associated with poor prognosis. Its pathogenesis has been shown to be correlated with JWA expression. JWA is also known as ADP ribosylation factor-like GTPase 6 interacting protein 5 (ARL6IP5) and works to protect the cells from DNA damage. Therefore, JWA is considered a tumor suppressor. In addition, JWA targeting has been proposed as a potential therapeutic strategy not only for GC but also for several other cancers [55]. In cancers, JWA is downregulated by degradation following its ubiquitination on lysine residues mediated by interaction with RNF185. High levels of RNF185 are correlated with shorter overall survival of GC patients. Furthermore, higher levels of RNF185 and the consequent lower levels of JWA are associated with cell migration and metastasis [56]. Thus, RNF185 may be considered a possible prognostic marker and a candidate target for GC treatment.

Moreover, RNF185 total serum cell-free DNA concentrations have been found higher in patients with renal cell carcinoma (RCC) than in patients with benign tumors. Patients with high RNF185 levels have a significantly worse prognosis, and a significant association of total cell-free RNF185 DNA levels with survival has been found, making RNF185 DNA a possible prognostic marker also in RCC [57].

RNF185 is an RNF5 homolog, and RNF5 has been found to exert pro- or anti-tumor activity depending on the tumor model. In neuroblastoma and in melanoma patients, RNF5 expression is correlated with better disease outcome. Analog-1 is a pharmacological activator of RNF5, and treatment of neuroblastoma and neuroblastoma cell lines reduces their viability. Furthermore, Analog-1 delays tumor growth in mouse models of neuroblastoma and melanoma [58]. Thus, RNF5 may represent a possible target for neuroblastoma and melanoma treatment, and considering the homology with RNF185, the latter ligase may represent a possible focus for a therapeutic strategy for these cancers as well.

**Figure 2:**
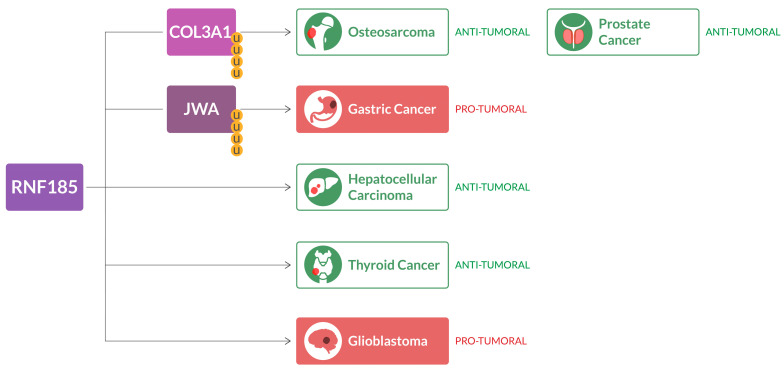
RNF185 in cancer. RNF185 ligase is downregulated in hepatocellular carcinoma and thyroid carcinoma. Through ubiquitination (shown as yellow U in the figure) of COL3A1, RNF185 plays a tumor suppressor role in osteosarcoma and prostate cancer. However, RNF185 could function as an oncogene in gastric cancer (via ubiquitination of JWA) and in glioblastoma.

Glioblastoma (GBM) is the most common primary brain tumor. It is a very aggressive cancer, and the prognosis remains poor as there is a lack of effective therapies. E3 ligases have been shown to play a key role in GBM pathogenesis. Thus, a research team has used the CRISPR system to downregulate E3 ligases in glioma cells. The gene analysis encouraged the researchers to further study RNF185. They found that in two glioma cell lines, this gene is a tumor suppressor. Further studies have shown that RNF185 downregulation is dependent on the promoter hypermethylation and increased levels of miR-587 [59]. Thus, E3 ligases such as RNF185 may represent a novel target for glioblastoma therapy either by reducing the promoter hypermethylation state or by modulating the miR-587 levels or both in combination therapy approaches (Figure 2).

## 4. MUL1

Mitochondrial Ubiquitin Ligase 1 (MUL1) is also a RING E3 ligase. It is located in the outer mitochondrial membrane (OMM), with its active RING domain facing the cytoplasm. This makes this mitochondrial E3 ligase capable of ubiquitinating cytosolic targets. It was first identified in 2008, and it is also known as MULAN (Mitochondrial Ubiquitin Ligase Activator of NFkB), MAPL (Mitochondrial Anchored Protein Ligase), GIDE (Growth-Inhibition and Death E3 Ligase) and HADES [60,61].

Other than its E3 ubiquitin ligase activity, MUL1 can also function as a SUMO E3 ligase, targeting different targets for SUMOylation [62]. This further broadens MUL1’s range of activities and molecular processes in which the ligase is involved, depending on the various targets of the ligase and the type of post-translational modification involved.

Known substrates of MUL1 include DRP1, MFN2, ULK1, Akt2, STING and p53. In addition, MUL1 has been linked to different cellular processes, such as mitochondrial dynamics, apoptosis, mitophagy, innate immune response and mitochondrial and cellular metabolism. MUL1 is mostly known for its involvement in mitophagy, where it can act independently of Parkin in order to regulate the process [63,64,65,66,67,68,69,70,71,72,73,74]. MUL1 can also regulate the mitophagy mediated by Parkin, by SUMOylating and activating the autophagic receptor NDP52, a well-known Parkin activator [75].

MUL1 can be regulated at the mitochondrial level by the action of the serine protease Omi/HTRA2, thus keeping its levels at a steady state [71]. MUL1 expression can also be regulated by AMPK activity [76]. In addition, MUL1 can regulate itself via auto-ubiquitination [77]. Overall, MUL1 dysregulations can impact several pathologies, such as inflammatory, cardiovascular and neurological diseases; MUL1 has been proposed as a potential therapeutic target, concerning especially the mitochondrial derangements that may occur during these diseases [78].

In the context of cancer, MUL1 can act both as an oncogene and as a tumor suppressor. The reason for this dualistic effect ultimately relies on the different targets of MUL1, which if dysregulated can lead to the activation of pro- or anti-tumoral pathways.

This is the case of p53, which gets directly ubiquitinated by MUL1, as seen in BC cells [79]. MUL1 is thus able to regulate p53 function, on a different lysine residue than the “canonical” one which is targeted by the Murine Double Minute 2 (MDM2) for p53 degradation [80]. MUL1 is able to co-localize with p53, thus blocking its ability to translocate and activate apoptosis, and to inhibit cancer growth. This has been observed also in lung carcinoma cells, where MUL1, with the same molecular mechanism, has been associated with the ubiquitination of p73, a structural and functional homolog of p53 [81]. These preliminary studies, which still need in vivo confirmation, suggest an oncogenic role for MUL1 in breast and lung cancer. Especially in lung cancer, MUL1 could have an oncogenic role: its levels can be induced by cigarette smoke, leading to alteration in lung vascular epithelial cells [82].

MUL1 has also been identified as a marker of poor prognosis for gliomas: although the molecular mechanisms are still to be identified, high expression of MUL1 in glioma patients is associated with lesser survivability [83,84].

Based on these reports, a therapy involving MUL1 inactivation, either by gene therapy or by developing a specific pharmacological inhibitor, could be useful to achieve cancer regression in breast cancer, lung cancer and glioma.

On the other hand, MUL1 has a tumor suppressor role in other types of cancers.

In fact, its absence leads to the accumulation of targets like Akt2, a well-known mediator of cancer cell survival [85], and to an overall higher activation of Akt [86]. This makes MUL1 an interesting target for a therapeutic strategy aimed at reducing the levels of Akt (and especially Akt2) in cancers that overexpress it such as breast, lung and colon cancer.

Furthermore, studies in ovarian cancer have indeed associated MUL1 induction by metformin with decreased Akt levels and cancer suppression [87]. Moreover, as shown in thyroid cancer cells, treatment with cisplatin was able to reduce Akt levels in a MUL1-dependent manner, and this mechanism is ultimately mediated by the action of FOXO3 [76].

Additionally, the ligase is seen inactivated in head-and-neck cancer: in this context, MUL1 induction by chemical treatment results in the ubiquitination of the heat shock protein 5 (HSP5), with consequent ER stress and autophagy/apoptosis induction [88]. All these data highlight a possible role for MUL1 in the treatment with chemotherapeutic drugs, as a mediator of a synergistic effect with traditional chemotherapeutic compounds. In this regard, MUL1 induction by gene therapy or by the inhibition of a regulator (such as Omi/HTRA2) would represent a valid therapeutic strategy.

MUL1 plays critical roles also in cellular metabolism regulation. As seen in HEK293 and Hela cells, inactivation of MUL1 results in a precise metabolic phenotype, due to the upregulation of direct targets such as Akt2 and indirect targets such as HIF-1α. [65] This metabolic phenotype is reminiscent of the Warburg effect, where oxidative phosphorylation (OXPHOS) is reduced and aerobic glycolysis is enhanced. Moreover, overexpression of MUL1 could lead to opposite results, by leading to increased OXPHOS [89]. Since metabolic alteration can be exploited by cancer cells in order to achieve survival and progression [10], MUL1 levels in cancer could be predictive of a possible metabolic switch. Furthermore, modulating its levels could be leveraged to target cancer metabolism for therapeutic purposes.

**Figure 3:**
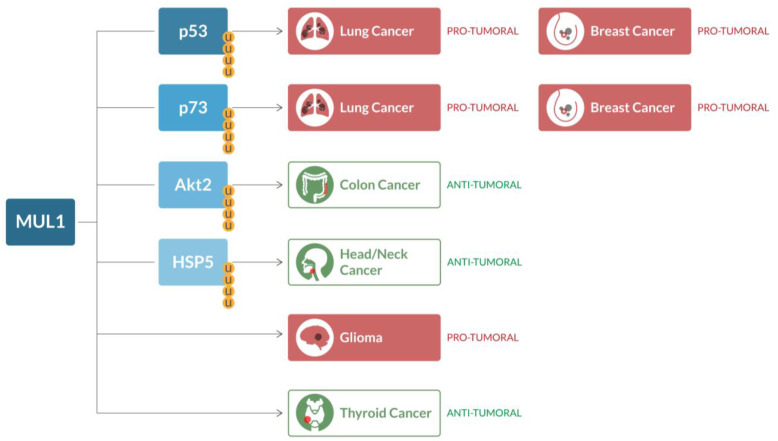
MUL1 in cancer. Depending on the cancer context, MUL1 acts as an oncogene in lung and breast cancer (through p53-p73 ubiquitination, shown as yellow U in the figure) and glioma (associated with lesser survivability). On the other hand, MUL1 acts as a tumor suppressor in colon cancer (through ubiquitination of Akt2), head-and-neck cancer (through ubiquitination of HSP5) and thyroid cancer.

The overexpression of both Akt2 and HIF-1α is related to cancer progression, besides metabolism [90,91]. Thus, monitoring MUL1 levels in those cancers that are known to overexpress these two factors may be predictive of a poor prognosis. In addition, a therapy aimed at restoring the levels of MUL1 in those cancers may downregulate Akt2 and HIF-1α and achieve tumor regression, as seen in other models [91]. MUL1 levels could also serve as a marker in those contexts, since it should be predictive not only of the cellular metabolism but also of the levels of its substrates (Figure 3).

## 5. Conclusions and Future Directions

The role of the mito E3 ligases in cancer is more complex than initially anticipated. As analyzed in this review, alterations, both positive and negative, in the ubiquitination and degradation of specific targets of the mito E3s contribute to cancer cell survival and cancer progression. Based on the reviewed literature, MARCH5 has mostly an oncogenic role, RNF185 acts as a tumor suppressor, whereas MUL1 can have both roles depending on the cell type.

On these bases, we believe that a targeted therapy approach based on the mito E3s in cancer could be effective to restore their function where they are inactivated or by reducing their levels where they are overexpressed. These effects could be achieved either by gene therapy or by the synthesis and use of specific activators and/or inhibitors of all three mito E3s.

However, one issue in this regard is that the involvement of some mito E3 in cancer has not yet been investigated, especially when they share a target of ubiquitination (such as MFN2, ubiquitinated by both MARCH5 and MUL1) or when they are associated with the same cellular process (such as the ERAD mechanism). Consequently, it is still unknown if the mito E3s act synergistically in the degradation of the common targets or if the enzymes compete for the ubiquitination reaction.

Moreover, from a therapeutic standpoint, the inhibition of a single mitoE3 could cause an increase in another one, in the context where two of them both act as oncogenes (for example, in breast cancer for MUL1 and MARCH5). In this case, a single inhibition therapy would be ineffective, and both ligases should be inactivated in order to achieve cancer regression. On the other hand, when two mito E3s act as tumor suppressors (such as in thyroid cancer for RNF185 and MUL1), induction of both enzymes could achieve a synergistic effect on cancer inhibition. However, all the possible interactions between the mito E3s remain to be investigated, in order to dissect the specific molecular mechanisms of activity.

A possible pathological overexpression of the mito E3 ligases could be countered by inhibiting the proteasome. As seen in multiple myeloma with the use of bortezomib and its derivates, the use of proteasome inhibitors (both alone and in combination with other compounds) leads to increased apoptosis, inhibition of angiogenesis and blockage of pro-tumoral cytokines and pathways [92]. Furthermore, resistance to proteasome inhibitors can be overcome using inhibitors of de-ubiquitinating enzymes (DUB), which act as antagonists of E3 ligases and may be upregulated in several cancers [93]. However, when considering the mito E3s, inhibition of the proteasome could represent a valid strategy only in those cancers where MARCH5, RNF185 and MUL1 are upregulated, with the objective to stop their excessive activity. In those tumors where the mito E3s act as tumor suppressors, the use of DUB inhibitors would be preferable.

Moreover, the role of all the mito E3 ligases in multiple myeloma (the cancer in which proteasome inhibitors are mostly used) remains to be investigated. For all these reasons, further research on the mito E3s in different tumors is mandatory, to further clarify their role as oncogenes or tumor suppressors, depending on the tumor types, and to highlight these enzymes as possible therapeutic targets in cancer.

## Data Availability

No new data were created or analyzed in this study. Data sharing is not applicable to this article.
